# Analysis of a Fiber-Coupled RGB Color Sensor for Luminous Flux Measurement of LEDs

**DOI:** 10.3390/s26020486

**Published:** 2026-01-12

**Authors:** László-Zsolt Turos, Géza Csernáth

**Affiliations:** Sapientia Hungarian University of Transylvania, Calea Sighișoarei 1c, 547367 Tirgu-Mures, Romania; csgeza@ms.sapientia.ro

**Keywords:** RGB photodiode sensor, luminous flux measurement, optical fiber coupling, LED metrology, spectral responsivity, uncertainty analysis, photometric calibration, optical head, white LED characterization, compact optical sensing system

## Abstract

Accurate measurement of luminous flux from solid-state light sources typically requires spectroradiometric equipment or integrating spheres. This work investigates a compact alternative based on a fiber-coupled RGB photodiode system and develops the optical, spectral, and geometric foundations required to obtain traceable flux estimates from reduced-channel measurements. The system under study comprises an LED with known spectral power distribution (SPD), optical head, optical fiber, a protective sensor window, and a photodiode matrix type sensor. A complete end-to-end analysis of the optical path is presented, including geometric coupling efficiency, fiber transmission and angular redistribution, Fresnel losses in the sensor window, and the mosaic structure of the sensor. Additional effects such as fiber–sensor alignment, fiber-facet tilt, air gaps, and LED placement tolerances are quantified and incorporated into a formal uncertainty budget. Using the manufacturer-supplied SPD of the reference LED together with the measured R, G, and B channel responsivity functions of the sensor, a calibration-based mapping is established to reconstruct photopic luminous flux from the three-channel outputs. These results demonstrate that, with appropriate modeling and calibration of all optical stages, a fiber-coupled RGB photodiode mosaic can provide practical and scientifically meaningful luminous-flux estimation for white LEDs, offering a portable and cost-effective alternative to conventional photometric instrumentation in mid-accuracy applications. Further optimization of computation speed can enable fully integrated measurement systems in resource-constrained environments.

## 1. Introduction

The accurate measurement of luminous flux from light-emitting diodes (LEDs) is fundamental in illumination engineering, solid-state lighting product development, and performance verification according to industrial and regulatory standards. While traditional methods for luminous flux determination rely on integrating spheres and calibrated photometers, several studies have explored whether spectral and photometric metrics can be derived from RGB or multi-channel sensors when appropriate calibration models are applied. As LED-based lighting systems proliferate in consumer electronics, automotive lighting, there is increasing interest in alternative flux-measurement techniques that are lower in cost, physically compact, and facilitating integration into automated test environments or embedded monitoring systems.

Reduced-channel spectral sensors, including RGB or multi-band photodiodes, have emerged as promising candidates for such applications. While these sensors cannot directly measure the full spectral power distribution (SPD) of a light source, a growing body of research has demonstrated that, when combined with appropriate calibration and modeling, their outputs can be transformed into photometric and colorimetric quantities with useful accuracy. Prior work has examined the use of RGB sensors for estimating illuminance, correlated color temperature (CCT), circadian metrics, and relative spectral characteristics of various light sources. However, significantly less attention has been paid to the problem of estimating absolute luminous flux of LEDs using RGB sensors, particularly in systems that include non-ideal optical components such as optical heads, fibers, protective windows, and mosaic sensor geometries.

## 2. Literature Review and Related Work

Studies such as Dias et al. [1] and Tran et al. [2] demonstrated that compact RGB-based systems can approximate both visual and non-visual lighting metrics with sufficiently low error for indoor monitoring and wearable-lighting applications. These systems typically combine RGB photodiodes with empirical or model-based transformations to estimate photometric quantities, showing that reduced-channel measurements can capture enough spectral information for practical applications when calibration is performed using a reference spectroradiometer. Vera-Duarte et al. [3] reported usage of plastic optical fiber coupled with an RGB photodiode array for colorimetric liquid analysis, revealing that the combination of fiber transport and reduced-channel detection can produce reliable color metrics when the optical chain is well characterized.

Other papers focus on calibration strategies for mapping RGB sensor outputs to photometric or spectral quantities. Lee et al. [4], for example, explored calibration of an RGB sensor combined with a white LED to improve measurement accuracy for chemical concentration detection. Their approach involved linearization, spectral correction, and modeling of sensor non-idealities.

Other relevant publications address traceable measurement techniques based on well-characterized LED spectral data. Kokka et al. [5] introduce an LED-based reference source, developed within the EMPIR PhotoLED framework, which is optimized for calibrating the spectral responsivity of photodetectors; their work analyzes spectral stability, drive-current effects, and temperature dependence. Zaid et al. [6] present traceable methods for the spectral characterization of solid-state lamps, including LEDs, with emphasis on accurate determination of spectral irradiance and luminous quantities, thereby complementing and supporting the use of the PhotoLED spectral database.

This paper investigates the feasibility of using a fiber-coupled RGB photodiode system for estimating the luminous flux of LEDs. A detailed characterization of the optical chain is performed. The analysis includes the geometric coupling behavior of the optical head, fiber transmission and angular redistribution effects governed by the fiber’s numerical aperture, Fresnel reflections and refraction at the sensor window, and the spatial sampling characteristics of the S9706 sensor. Effects due to fiber-end facet tilt, small air gaps between fiber and sensor, and positional tolerances in LED placement are similarly quantified. All relevant contributions are incorporated into a formal measurement uncertainty budget.

Using the known SPD of the reference LED and the measured spectral responsivity functions of the R, G, and B channels, a calibration-based mapping is established to transform the three-channel sensor outputs into an estimate of photopic luminous flux, demonstrating that a fiber-coupled RGB photodiode mosaic can serve as a compact and cost-effective tool for mid-accuracy luminous flux measurement in applications where portability, integration, or low cost are prioritized over high-end photometric precision. The remainder of this paper presents the detailed system characterization, calibration methodology, uncertainty analysis, and validation measurements supporting this conclusion.

## 3. Sensor Selection

Alternative color and multispectral sensors were evaluated, including the ams-OSRAM TCS3701 and TCS3707 (ams-OSRAM AG, Premstaetten, Austria) devices, the AS7262, AS7263, and AS7341 multispectral arrays, and several Hamamatsu photodiodes, such as the S9706, S9705, S9704, S9032, and S9133 (HAMAMATSU PHOTONICS K.K., Solid State Division, Hamamatsu City, Japan) series. These sensors offer more advanced functionality than single-pixel RGB detectors. The goal was to identify those that provide the required combination of spatial sampling, large-area photodiodes, and hardware-parallel channel integration necessary for high-accuracy flux measurement in a fiber-coupled configuration.

The TCS3701 and TCS3707 implement small multi-zone photodiode structures intended to improve angular response, but they do not form a true mosaic array, and their total active area remains too small for efficient coupling to a 0.5 mm optical fiber. Their sequential channel readout also prevents simultaneous R, G, and B integration, limiting their suitability for measurements sensitive to LED warm-up effects or temporal variations.

The AS7262, AS7263, and AS7341 multispectral sensors provide multiple narrowband channels but rely on very small individual photodiodes with low spatial redundancy. Their optical design targets free-space illumination and offers limited compatibility with direct fiber coupling. The time-multiplexed acquisition of their spectral bands further increases susceptibility to transient or temperature-dependent LED behavior.

Hamamatsu devices, including the S9706, S9705, S9704, S9032, and S9133 families, incorporate segmented or multi-zone photodiode structures intended for precise optical power or colorimetric measurement. Among these, the S9706 [7] exhibits substantially higher segmentation density and total active area, providing improved spatial uniformity, pixel count, and parallel color-channel integration. These characteristics make the S9706 the most suitable sensor for meeting the stringent requirements of a fiber-coupled luminous-flux measurement system.

## 4. System Description

**Figure 1:**
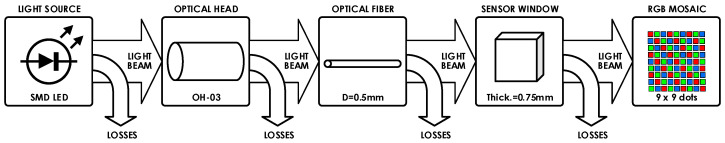
Optical path of the system.

The system consists of five principal components (Figure 1) arranged in series: (1) the LED under test with known spectral power distribution (SPD), (2) an OH-03 optical capture head (OCH, Feasa Enterprises Limited, Limerick, Ireland), (3) a 0.5 mm diameter, widely adopted, low-bending-radius polymer optical fiber (POF), (4) the Hamamatsu S9706 RGB sensor’s 0.75 mm protective glass window, and (5) the Hamamatsu S9706 RGB sensor’s photodiode mosaic. Each component introduces wavelength-dependent transmission, geometric filtering, or spatial sampling effects that collectively define the end-to-end system response.

### 4.1. LED Source and Spectral Model

The test source is a phosphor-converted white LED (LUXEON 3014, 4000 K, LUMILEDS Netherlands B.V., Eindhoven, The Netherlands), selected for its well-characterized spectral power distribution provided by the manufacturer. The spectral radiant flux of the LED is modeled as:

(1) $$\Phi_{e}\left(\lambda \right)=P_{e}\;S_{\mathrm{LED}}\left(\lambda \right)$$

where $S_{\mathrm{LED}}\left(\lambda \right)$ is the normalized SPD and $P_{e}$ is a scaling factor determined by the LED’s drive current and known luminous flux specification.

The photopic luminous flux is defined by:

(2) $$\Phi_{v}=K_{m}\int_{\lambda }\Phi_{e}\left(\lambda \right)V\left(\lambda \right)d\lambda ,$$

where $V\left(\lambda \right)$ is the CIE 1931 photopic sensitivity function and $K_{m}$ is the maximum luminous efficacy at 555 nm.

(3) $$K_{m}=683\mathrm{lm}W^{-1}$$

Since the RGB sensor does not measure $\Phi_{e}\left(\lambda \right)$ directly, reconstructing $\Phi_{v}$ requires developing an optical transfer function linking the LED SPD to the sensor outputs.

### 4.2. Optical Capture Head

The optical head is designed to couple a controlled fraction of the LED’s emission into an optical fiber. It acts as a geometric sampler, imposing constraints on both the acceptance angle and the spatial region from which light is collected. The head exhibits less than 10% sensitivity variation for a ±1 mm displacement of the LED, indicating moderate tolerance to placement errors.

The coupling efficiency $k_{\mathrm{OCH}}$ is modeled as:

(4) $$k_{\mathrm{OCH}}=\frac{\Phi_{e,\mathrm{captured}}}{\Phi_{e,\mathrm{LED}}}$$

and depends on LED position, its emission profile, and the head’s optical acceptance. Under fixed mechanical alignment, $k_{\mathrm{geo}}$ is treated as constant but contributes to the overall measurement uncertainty.

### 4.3. Optical Coupling Between Polymer Optical Fiber and RGB Sensor

The polymer optical fiber (POF) used in this work has a core diameter of 0.5 mm and guides light by total internal reflection. The angular distribution of the emerging light is governed by the numerical aperture (NA) of the fiber. The maximum exit half-angle in air is given by

(5) $$\theta_{\mathrm{air}}=\arcsin\left(NA\right)$$

For typical POFs with NA≈0.5, this yields $\theta_{\mathrm{air}}\approx 30^{\circ },$ corresponding to a full output cone angle of approximately $60^{\circ }$ [8].

The Hamamatsu S9706 sensor by construction includes a flat glass window of thickness *t* = 0.75 mm and refractive index $n_{\mathrm{glass}}\approx 1.5$ protecting the sensor area. When the divergent beam enters the glass from air, refraction occurs according to Snell’s law:

(6) $$n_{\mathrm{air}}sin\;\theta_{\mathrm{air}}=n_{\mathrm{glass}}sin\;\theta_{\mathrm{glass}}$$

which reduces the propagation angle inside the glass to:

(7) $$\theta_{\mathrm{glass}}=arcsin\;\left(\frac{n_{\mathrm{air}}}{n_{\mathrm{glass}}}sin\;\theta_{\mathrm{air}}\right)\approx 19.5^{\circ }$$

At these moderate incidence angles, Fresnel reflection losses at the air–glass interface are small and are neglected in the first-order analysis. The radial expansion of the beam as it propagates through the glass window is given by:

(8) $$\Delta r=ttan\theta_{\mathrm{glass}}\approx 0.75\cdot tan(19.5^{\circ })\approx 0.27\;\mathrm{mm}.$$

Assuming that the fiber end face is in direct contact with the sensor glass, the effective beam radius at the photosensitive surface can be approximated as:

(9) $$r_{\mathrm{spot}}=\frac{d_{\mathrm{fiber}}}{2}+\Delta r$$

**Figure 2:**
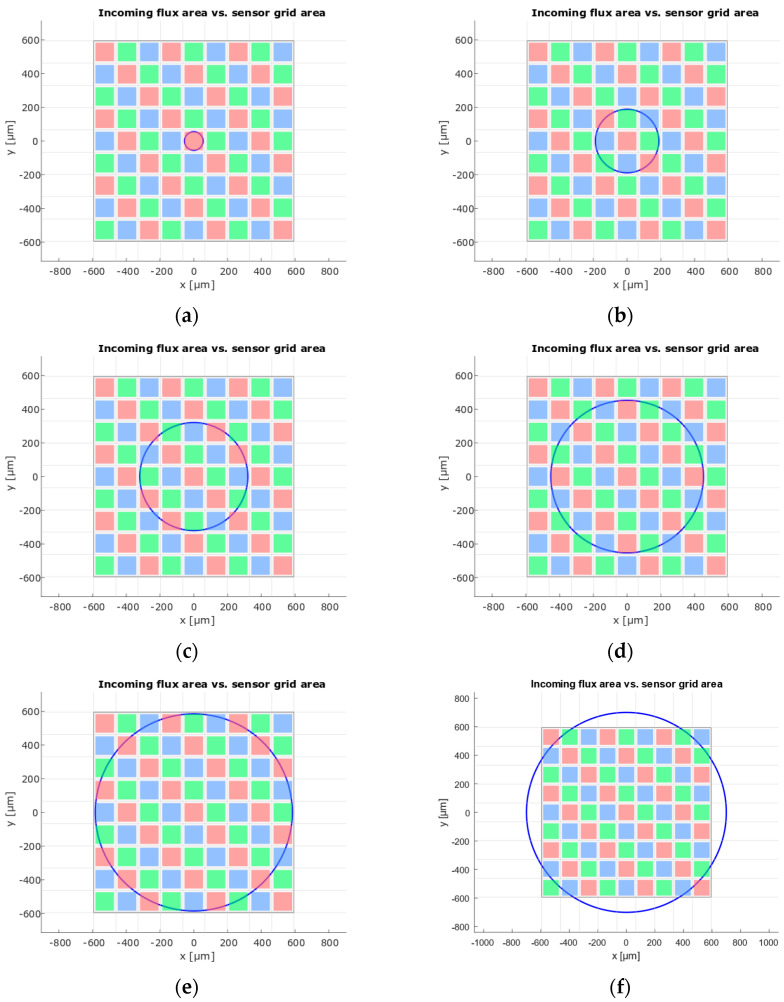
Incoming flux area (interior of the circle) and active sensor area (R, G, B colored minus white area), when the light spot size matches: (**a**) one single sensing dot−efficiency is 100%; (**b**) roughly 9 sensing dots−efficiency is 74%; (**c**) more than 16 sensing dots−efficiency is 71%; (**d**) 75% of the entire sensing area−efficiency is 71%; (**e**) up to 92% of the entire sensing area−efficiency is 70%; (**f**) spot border is beyond the sensing area−efficiency is 60%.

With $d_{\mathrm{fiber}}=0.5\;\mathrm{mm}$, this gives $r_{\mathrm{spot}}=0.52\;\mathrm{mm},$ corresponding to an illuminated spot diameter of approximately 1.04 mm. This spot size is comparable to the photosensitive area of the S9706 sensor, fitting between the areas represented in Figure 2d,e.

### 4.4. Sensor Window and Fresnel Effects

A 0.75 mm glass window covers the RGB photodiode mosaic. The window introduces two air–glass interfaces, each contributing Fresnel reflection of approximately 4% at normal incidence. These losses are treated as wavelength-independent in the visible range and combined into a single transmission factor:

(10) $$T_{\mathrm{glass}}\approx (1-R{)}^{2}\approx 0.92$$

where R≈0.04. Minor angular refraction effects are negligible due to the small fiber–window spacing and the approximately collimated nature of the fiber output.

### 4.5. RGB Photodiode Mosaic and Flux-Transfer Analysis

The S9706 detector (Hamamatsu) comprises a 9 × 9 mosaic (81 sub-pixels, Figure 2a) with color filters arranged in an RGB pattern. Each sub-pixel consists of a 110 µm × 110 µm active area surrounded by a light-shielded border, yielding a 132 µm pitch. Due to the finite output cone of the 0.5 mm diameter POF, the emitted beam forms an illuminated spot with an area of approximately 0.849 mm^2^ at the sensor surface. The Hamamatsu S9706 RGB sensor has a total geometric photosensitive area of 1.411 mm^2^; however, only 0.980 mm^2^ of this area is actively photosensitive, as the individual sensing elements are separated by reflective white corridors. This results in an effective sensing fill factor of 69.4%. To account for this geometrical mismatch a geometrical coupling efficiency, defined as the fraction of incident flux that actually reaches active photodiode areas, is introduced:

(11) $$k_{\mathrm{SNS}}\approx 0.694$$

Since the optical spot overlaps a sensor surface that is not fully covered by active sensing elements, only a fraction of the incident luminous flux is intercepted and converted into an electrical signal:

(12) $$\Phi_{e}=k_{\mathrm{SNS}}\;\Phi_{\mathrm{incident}}$$

The photocurrent in each color channel is given by:

(13) $$I_{R}=\int_{\lambda }\Phi_{e}\left(\lambda \right)\;T_{\mathrm{fiber}}\left(\lambda \right)\;T_{\mathrm{glass}}\;S_{R}\left(\lambda \right)\;A_{\mathrm{active}}\;d\lambda$$

with analogous expressions for the G and B channels. Here: $S_{R}\left(\lambda \right)$, $S_{G}\left(\lambda \right)$, and $S_{B}\left(\lambda \right)$ are the spectral responsivity functions of the R, G, and B sub-pixels.

The sensor outputs are digitized with 12-bit resolution through a 36-bit (12 bits × 3 channels) serial readout. Depending on the expected illumination level, the optical system can be designed to utilize one of the remaining radii—188 µm, 320 µm, 452 µm, or 584 µm—to achieve an optimal balance between sensitivity and dynamic range. Under low-light conditions, a radius of 584 µm is recommended to ensure that all three channels (R, G, B) generate sufficiently large signals. Conversely, in high-illumination conditions, a radius of 188 µm is preferable to prevent saturation of the RGB outputs.

**Figure 3:**
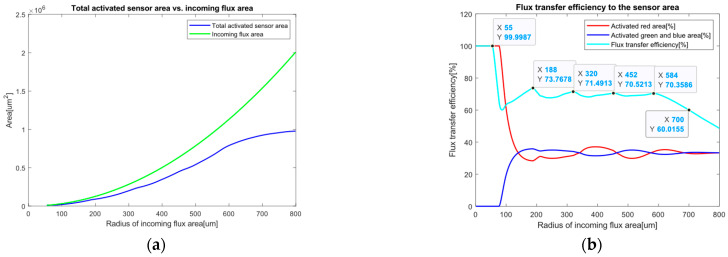
(**a**) Total activated sensor area versus incoming flux area; (**b**) flux transfer efficiency with local maximums.

The flux-transfer analysis reveals spatially periodic variations arising from the pixel mosaic structure of the S9706. Several local maxima appear in the flux transfer efficiency diagram η(r), Figure 3b, each corresponding to a specific radius (r) at the base of the light cone entering the sensor’s active area. These radii occur at 55 µm, 188 µm, 320 µm, 452 µm, and 584 µm. Although the efficiency reaches 100% at 55 µm, this value is not practical because, at that radius, only the photodiodes sensitive to the red spectrum S_R_(λ) contribute to the measured flux. The activated-area functions for green and blue pixels overlap exactly due to their symmetric placement, whereas the red-versus-green/blue curves are nearly anti-phase, with intersection points nearly coinciding with local maxima in transfer efficiency. For larger radii the efficiency tends asymptotically towards the mosaic active-area fraction (~69.4%), so the operating range of the actual system, with a fiber footprint radius of approximately 250 µm, lies in the regime where η(r) is close to this asymptotic value but still exhibits weak oscillatory modulation due to the underlying pixel geometry, which must be considered in the calibration and uncertainty analysis.

The S9706 sensor also includes a digital control input that allows selection of either the full active area (9 × 9 array, 81 photodiodes) or only the central region (3 × 3 array, 9 photodiodes), providing further flexibility in adapting to different illumination levels.

### 4.6. End-to-End Optical Transfer Function

Combining all components, the system’s effective spectral responsivity is:

(14) $$R_{\mathrm{sys},c}\left(\lambda \right)=k_{\mathrm{OCH}\;}k_{\mathrm{SNS}\;}k_{\exp}\;T_{\mathrm{fiber}}\left(\lambda \right)\;T_{\mathrm{glass}}\;A_{\mathrm{active}}\;S_{c}\left(\lambda \right),c\in \left\{R,G,B\right\}$$

From (8) a K factor can be defined as:

(15) $$K=k_{\mathrm{OCH}\;}k_{\mathrm{SNS}\;}k_{\exp}\;T_{\mathrm{fiber}}\left(\lambda \right)\;T_{\mathrm{glass}}\;A_{\mathrm{active}}$$

where K includes amplification, integration time, conversion gain and sensing area. This set of equations forms the basis of the calibration and inversion procedure used to reconstruct photopic luminous flux from the RGB measurements.

## 5. Flux Calculation Methods

Using RGB color sensors for luminous flux measurement is not straightforward. An RGB sensor does not measure directly the spectral radiant flux $\Phi_{e}(\lambda )$ and its sensitivity is different from the human eye photopic sensitivity $V\left(\lambda \right)$. The incident light signals detected by the photodiodes are amplified and converted into digital signals. Each R, G and B element usually has an on-chip filter that is more sensitive to one color of light, either red (λ_PR_ = 615 nm), green (λ_PG_ = 540 nm) or blue (λ_PB_ = 465 nm). Thus, the RGB sensor measures three discrete values:

(16) $$R=K\int_{\lambda }S_{R}\left(\lambda \right)\;\Phi_{i}\left(\lambda \right)d\lambda$$

(17) $$G=K\int_{\lambda }S_{G}\left(\lambda \right)\;\Phi_{i}\left(\lambda \right)d\lambda$$

(18) $$B=K\int_{\lambda }S_{B}\left(\lambda \right)\Phi_{i}\left(\lambda \right)d\lambda$$

where $\Phi_{i}\left(\lambda \right)$ is the incoming radiant flux received from the sensor and $S_{R}(\lambda )$, $S_{R}(\lambda )$, $S_{R}(\lambda )$ the sensor spectral sensitivity curves and λ_PR_, λ_PG_, λ_PB_ are peak values of these curves where they are most sensitive. Generally, in order to compute luminous flux the input data which is needed are the sensor’s spectral sensitivity curves $S_{R}(\lambda )$, $S_{R}(\lambda )$, $S_{R}(\lambda )$, the human eye photopic sensitivity curve $V\left(\lambda \right)$, the R,G,B data outputted from the sensor which has to be weighted with the exposure time (the length of time the sensor is exposed to incoming light) and the sensor’s active (sensitive) area which are sensitive to different wavelengths of the light.

### 5.1. Method for Relative Luminous Flux

When relative luminous flux is sufficient and absolute values are not needed one can use the following formula with the CIE standard coefficients.

(19) $$\Phi_{n1}=0.2126R_{n}+0.7152G_{n}+0.0722B_{n}$$

where $R_{n},G_{n},\;B_{n}$ are the normalized R, G, B values between 0…1.

Thus, for an *n* bit resolution RGB sensor, in order to obtain $R_{n},{\;G}_{n}$, $B_{n}$ the R, G, B values measured by the sensor should be divided by $2^{n-1}$. This weighted sum will give an approximate value not accurate for real sensors, calibration could help.

### 5.2. Method by Solving a Linear System and Using Calibration

On of the easiest and practical method is to solve a linear system in order to approximate the luminous flux:

(20) $$\Phi_{v}=k_{R}R+k_{G}G+k_{B}B$$

where $k_{R},k_{G},k_{B}$ are coefficients that either are given in the sensor’s datasheet or these parameters can be obtained experimentally and by calibration by using a reference spectrometer or fluxmeter and performing multiple measurements for known light sources and also at different flux values of the same light source. In case coefficients are not available from datasheet an optimal solution can be found by using least square method.

### 5.3. Method Using Sensor Spectral Sensitivity Curves Combined with Photopic Weighting

**Figure 4:**
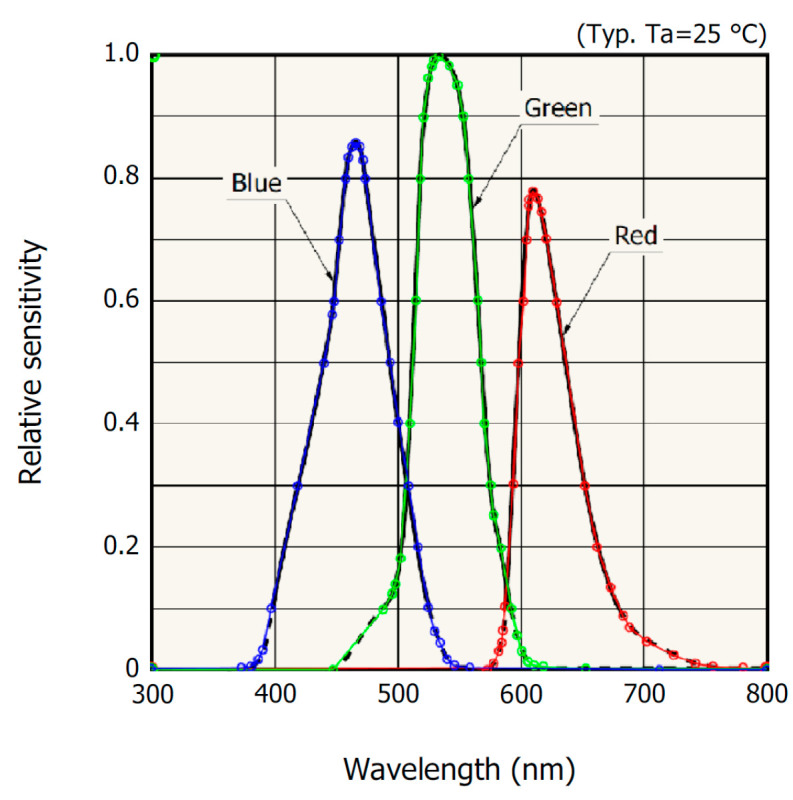
The digitization process of spectral sensitivity from the S9706 sensor datasheet.

The sensor datasheet provides the spectral sensitivity curves $S_{c}\left(\lambda \right),$ $c\in \left\{R,G,B\right\}$. These curves were digitized using custom software developed in the MATLAB (ver. R2023b) environment to facilitate their extraction and further processing. The digitization procedure began with defining the coordinate system by selecting two reference points on the *x*-axis (300 nm and 800 nm) and on the *y*-axis (0 and 1). Next, a series of representative points were manually selected along each of the S_R_, S_G_, S_B_ curves. After collecting these points, interpolation was applied to reconstruct the continuous spectral sensitivity functions (Figure 4).

The CIE photopic sensitivity data $V\left(\lambda \right)$ can also be obtained from publicly available sources and processed to match the wavelength interval used for the sensor spectral sensitivity. To ensure compatibility, the photopic curve was reproduced, resampled, and adjusted to the same 300–800 nm range used for the sensor. The sensor’s spectral sensitivity curves were likewise normalized.

**Figure 5:**
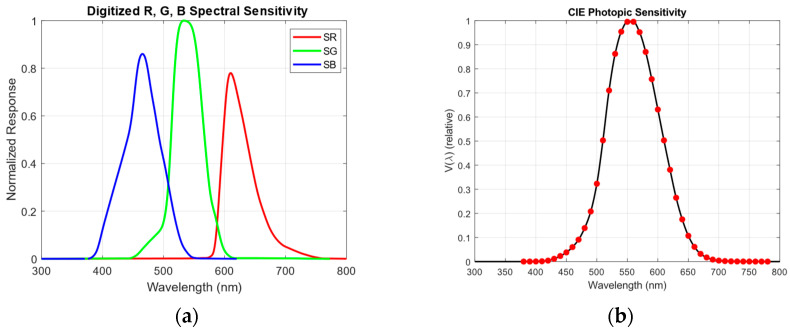
Spectral sensitivity graphs: (**a**) R, G, B spectral sensitivity of S9706; (**b**) CIE photopic sensitivity of human eye.

Figure 5a,b show the resulting normalized and digitized spectral sensitivity functions (S_R_(λ), S_G_(λ), S_B_(λ)) extracted from the Hamamatsu S9706 sensor datasheet, together with the processed photopic sensitivity curve. Because the CIE standard defines photopic data only over the 380–780 nm interval, the data were extrapolated to the full 300–800 nm range to match the wavelength coverage of the sensor.

In order to find the normalized coefficients $k_{nR},k_{nG},k_{nB}$, compute:

(21) $$k_{c}=\int_{\lambda }V\left(\lambda \right)S_{c}\left(\lambda \right)d\lambda ,c\in \left\{R,G,B\right\}$$

Then calculate:

(22) $$\Phi_{v}=K\left(k_{R}R+k_{G}G+k_{B}B\right)$$

where K includes amplification, integration time (exposure time), and conversion gain. When the relative luminous flux is sufficiently high, the normalized coefficients can be determined as follows:

(23) $$k_{nc}=\frac{k_{c}}{k_{R}+k_{G}+k_{B}}c\in \left\{R,G,B\right\}$$

The obtained normalized $k_{nc}$ parameters form the final equation for the S9706 sensor after performing the algorithm.

(24) $$\Phi_{n3}=0.2034R_{n}+0.6822G_{n}+0.1144B_{n}$$

The values obtained show similarity to the values in Equation (19) as discussed in method Section 5.1, however these values are calculated exactly based on the sensor sensitivity correlated with the sensitivity of the human eye, which offer better results.

### 5.4. Method by Spectral Curve Reconstruction

**Figure 6:**
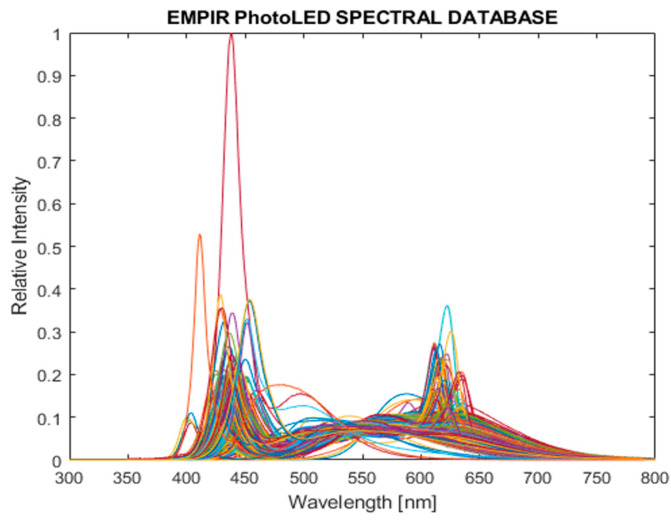
Representation of the normalized photoLED spectral database from 300 to 800 nm.

This approach represents the most complex method for estimating luminous flux. A fundamental challenge is the phenomenon of metamerism, whereby different spectral power distributions can produce identical R, G, and B sensor outputs. The first step in the method is to obtain the sensor’s spectral sensitivity functions $S_{c}\left(\lambda \right)$, $c\in \left\{R,G,B\right\}$. The procedure also requires a large input database of reference spectra with known radiometric properties. For this purpose, spectral datasets were obtained from the publicly available EMPIR PhotoLED database [9] and processed in MATLAB as shown in Figure 6.

For each reference spectrum in the database, the corresponding theoretical R, G, and B channel responses are computed as:

(25) $$R=\int_{\lambda }S_{R}\left(\lambda \right)\;\Phi_{ref}\left(\lambda \right)d\lambda$$

(26) $$G=\int_{\lambda }S_{G}\left(\lambda \right)\Phi_{ref}\left(\lambda \right)d\lambda$$

(27) $$B=\int_{\lambda }S_{B}\left(\lambda \right)\Phi_{ref}\left(\lambda \right)d\lambda$$

where $S_{c}\left(\lambda \right)$ $c\in \left\{R,G,B\right\}$ denotes the spectral responsivity of channel c and $\Phi_{\mathrm{ref}}\left(\lambda \right)$ is the spectral radiant flux of the reference LED.

Based on the resulting RGB spectrum mapping, a predictive model can be constructed, for example, by applying linear regression to determine a 3×n transformation matrix that best maps the RGB values to the spectra in the database. Alternatively, more advanced approaches such as deep learning may be employed.

**Figure 7:**
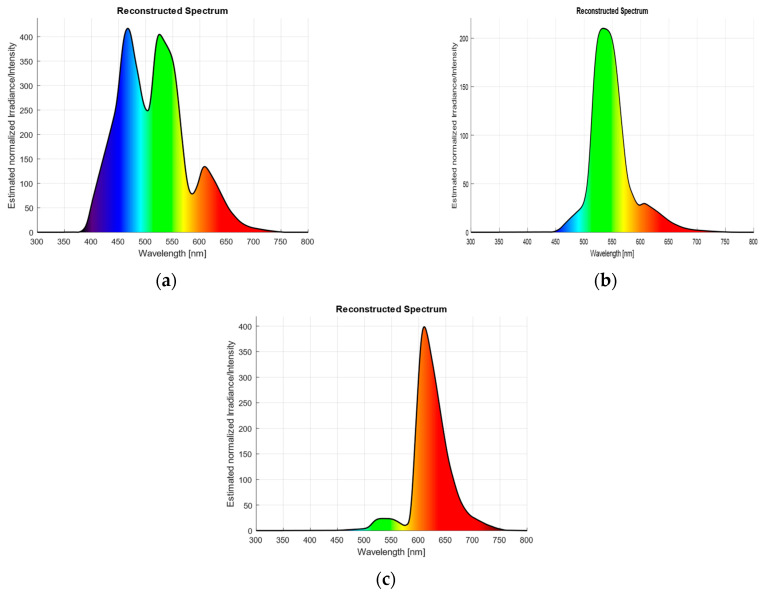
Reconstructed spectral graphs for: (**a**) cyan LED (R = 1354, G = 3007, B = 3844); (**b**) green LED (R = 612, G = 3604, B = 2); (**c**) red LED (R = 3961, G = 170, B = 1).

After the model is trained, a newly measured RGB triplet is supplied to the model to reconstruct an estimated spectral power distribution (eSPD) $P_{e}\left(\lambda \right)$, representing the most likely spectrum consistent with the training data. Once the eSPD is obtained, the photopic luminous flux can be calculated using

(28) $$\Phi_{v}=K_{m}\int_{\lambda }P_{e}\left(\lambda \right)\;V\left(\lambda \right)d\lambda$$

where $K_{m}$ is the maximum luminous efficacy and $V\left(\lambda \right)$ is the CIE photopic luminous-efficiency function. Measurements were performed for three colored LEDs, and the corresponding reconstructed spectral distributions are shown in Figure 7a for cyan, Figure 7b for green, and Figure 7c for red.

### 5.5. Comparison of Methods in Section 5.1 and Section 5.3

**Figure 8:**
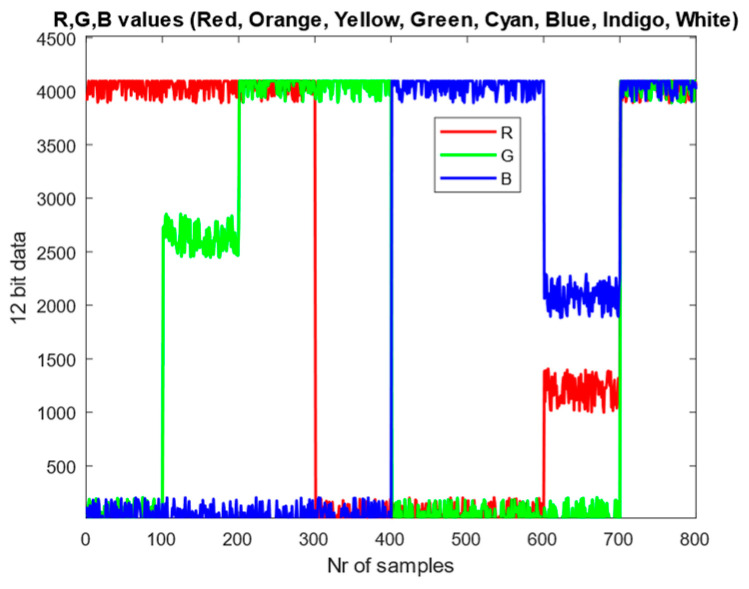
RGB data for colored LEDs with 10% spread.

To enable comparison, the final normalized equations were simulated for both methods. The R, G, and B values were combined to generate specific LED colors. A random ±10% noise was added around the ideal 12-bit R, G, and B values to emulate measurement noise from the sensor, after which the values were normalized. Each defined color set contains 100 samples, and in total eight types of LEDs were simulated (800 samples) in the following order: red, orange, yellow, green, cyan, blue, indigo, and white. The simulated data are shown in Figure 8.

**Figure 9:**
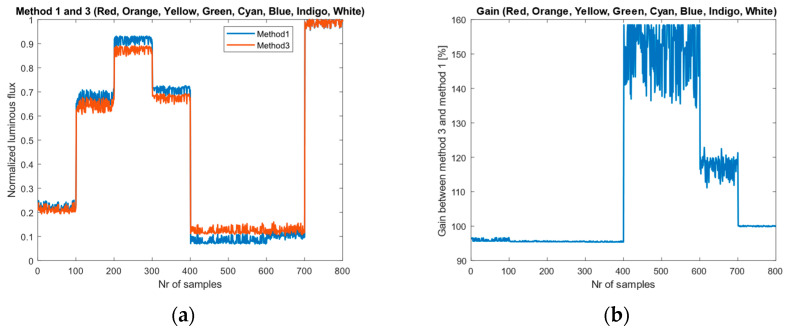
Comparison results: (**a**) Normalized luminous flux values; (**b**) Gain between method 1 and method 3.

The simulated normalized luminous-flux results are shown in Figure 9a. Since the absolute luminous flux is typically obtained by multiplying the normalized value by a constant that accounts for optical transfer efficiency, sensor active area, exposure time, and related factors, our primary interest is to determine the gain error (in percent) between the two methods. The gain is computed using the result of Method 1 as the reference:

(29) $$G=100\times \frac{\Phi_{n3}}{\Phi_{n1}}\left[\%\right]$$

As shown in Figure 9b, for the red, orange, yellow, green, and white LEDs, the gain remains within approximately 95–100%. In contrast, for indigo LEDs the gain increases to 110–120%. The highest gain is observed for cyan and blue LEDs, where the values range from 130% to 160%. These results indicate that using standard CIE color-matching functions together with real RGB sensors can produce significant errors, particularly for LEDs with certain spectral characteristics. Calibration can mitigate these discrepancies, but it must be performed separately for each class or type of LED.

## 6. Measurement, Data Extraction and Repeatability Analysis

### 6.1. Measurement and Data Extraction

For extracting measurement data from the system, the S9706 RGB photodiode sensor is interfaced to a generic 8-bit microcontroller that executes a dedicated firmware for data acquisition [10]. The microcontroller controls the sensor’s sensitivity setting via the Range (RNG.) input and sets the integration (exposure) period through the Gate signal. A clock signal (CLK) of up to 2 MHz is supplied to the sensor to synchronize serial readout of the three 12-bit channel values (36 bits total) following completion of the integration interval.

**Figure 10:**
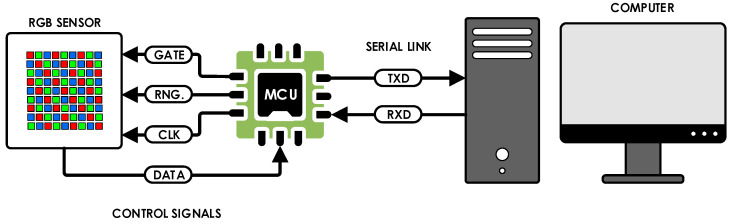
Block diagram of the measurement data extraction path from sensor to host computer.

After each measurement cycle, the acquired 36-bit data word is stored in a local buffer within the microcontroller’s data memory. The buffered results are then transmitted to a host computer over a standard UART interface (TXD and RXD lines). On the host side, the data stream is captured and organized into MATLAB data structures for subsequent processing, calibration, and analysis. The overall data-flow architecture and signal relationships are illustrated in Figure 10, which presents the measurement chain in block-diagram form.

**Figure 11:**
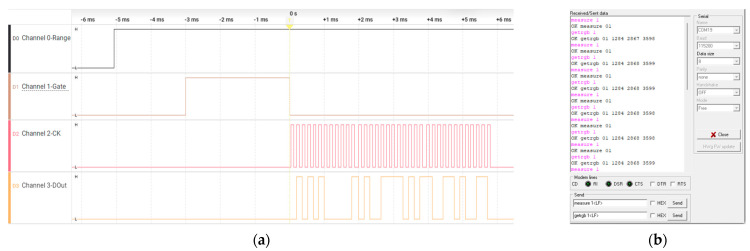
Communication details: (**a**) Captured signaling between MCU and sensor; (**b**) Terminal window sample with RGB data chain (MCU and PC).

The timing diagram of the data transfer between the sensor and the MCU reveals the simplicity of the signaling system. The minimal waiting time between consecutive reads of the RGB data fields is determined by the exposure time (Figure 11a—Gate signal). The RGB data collected by the MCU are retrieved by the PC using a simple serial protocol, which can be observed in the console window.

### 6.2. Repeatability and Temperature Stability

To evaluate measurement repeatability and temperature stability, consecutive measurements were performed using a human-readable ASCII serial protocol to trigger data acquisition and read out the results from the controller unit. To ensure sufficient signal levels (above 80% of the full-scale 12-bit RGB output), the exposure time (referred to as gating time in the datasheet) was set to 250 ms. A total of 100 samples were recorded over approximately 30 s, including communication overhead. The overall measurement speed is therefore primarily limited by the exposure time, especially under low-luminance conditions. In such cases, it is advantageous to maximize the effective sensing area to integrate more incident flux, thereby reducing the required exposure time to reach high-resolution RGB values.

**Figure 12:**
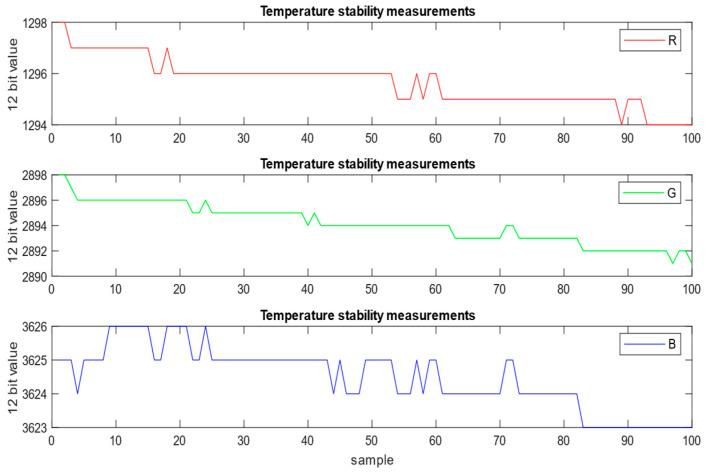
Measurement repeatability and temperature stability.

As shown in Figure 12, the raw 12-bit R, G, and B measurements exhibit good repeatability with only minor variations, typically limited to the least significant bits. However, a slow monotonic decrease can be observed over time: the R channel decreases from 1298 to 1294, the G channel from 2898 to 2890, and the B channel from 3626 to 3623 over the 30 s acquisition window. This behavior is attributed to the temperature dependence of the LED, where increasing junction temperature reduces quantum efficiency, resulting in lower emitted radiant flux. For this reason, in practical industrial applications it is recommended to perform luminous flux measurements shortly after LED turn-on to minimize thermal drift effects.

**Figure 13:**
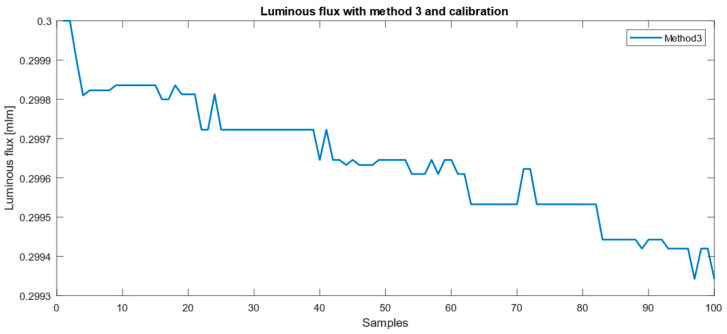
Drift tendency of the obtained luminous flux values.

The luminous flux calculated using Method 3, applying a calibration gain of 0.4626 derived from a 0.3 mlm reference measurement obtained with a spectrometer (Broadcom AFBR-S20M2VI, Broadcom Inc., Palo Alto, CA, USA), is shown in Figure 13.

Despite the observable temperature-induced drift, the calculated luminous flux remains within ±0.1% of the 0.3 mlm reference value throughout the measurement interval, demonstrating good short-term stability and repeatability of the proposed method.

## 7. Conclusions

This work investigated the feasibility of estimating LED luminous flux using a fiber-coupled RGB color sensor and analyzed the principal physical mechanisms governing the performance of such a measurement chain. A survey of relevant literature and commercially available sensors was conducted, leading to the selection of the Hamamatsu S9706 as the only device offering the required combination of large sensing area, pixel mosaic structure, and hardware-parallel color channel integration. A detailed optical system description was developed, covering the LED spectral model, the capture head, fiber transmission and angular redistribution, Fresnel effects at the sensor window, and the spatial sampling characteristics of the RGB photodiode array.

The geometric flux-transfer analysis revealed spatially periodic variations caused by the mosaic layout of the S9706, demonstrating that the effective light-collection efficiency depends on the beam footprint at the sensor surface. Several approaches for luminous flux estimation were examined, including relative-flux methods, calibration based linear systems, sensor spectral sensitivity curves combined with photopic weighting and a reconstruction approach that estimates the spectral power distribution from RGB measurements using reference spectral datasets. Simulations were performed to compare these methods under controlled conditions using color dependent noise models. A measurement and data extraction path were also implemented, providing a practical framework for future physical validation.

Although the present study does not yet constitute a full metrological validation against traceable standards, the experimental results demonstrate that the proposed fiber-coupled RGB photodiode approach can achieve stable and repeatable luminous-flux measurements with good agreement to a calibrated reference. The combination of optical modeling, geometrical coupling analysis, and experimental calibration confirms that the system is capable of estimating luminous flux with an accuracy better than 0.1% under controlled conditions. The observed temperature-dependent drift highlights the importance of thermal stabilization and timing considerations, particularly when measuring low-power LEDs. Overall, the results validate the feasibility of the proposed measurement concept and demonstrate that, with appropriate calibration and knowledge of the spectral power distribution, a compact RGB-sensor-based system can provide reliable luminous-flux estimation. The presented methodology establishes a solid experimental and analytical foundation for further refinement, including improved thermal management, broader spectral validation, and extension to LEDs with more complex or non-standard emission spectra.

## Data Availability

The original contributions presented in this study are included in the article. Further inquiries can be directed to the corresponding author.
